# Supplementary material for: The Impact of Nano- and Micro-Silica on the Setting Time and Microhardness of Conventional Glass–Ionomer Cements
Source: Dent J (Basel). 2024 Feb 27;12(3):54. doi: 10.3390/dj12030054 (PMC10968912; doi:10.3390/dj12030054)
Supplement: Supplementary file 1 [file dentistry-12-00054-s001.zip › dentistry-2819448-supplementary.pdf]

S.1. Microhardness

Representative example images of the measurement of the indentation for the evaluation of microhardness of KM and FIX are given in Figures S1 and S2.

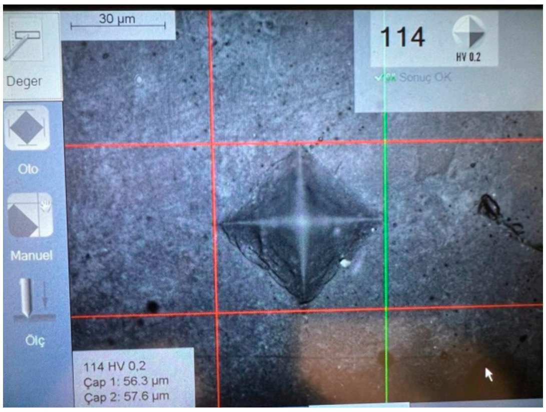

Figure S1. Measurement of indentation of KM.

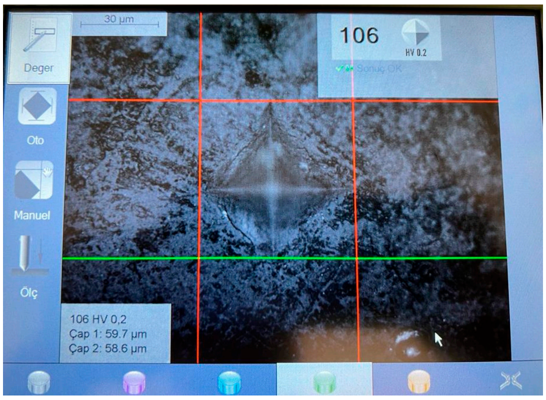

Figure S2. Measurement of indentation of FIX.

S.2. EDX Analysis

EDX spectra of KM incorporating 0 and 6 wt% nano- or micro-silica are presented in Figures S3-S5, and EDX spectra of FIX incorporating 0 and 6 wt% nano- or micro-silica are presented in Figures S6-S8.

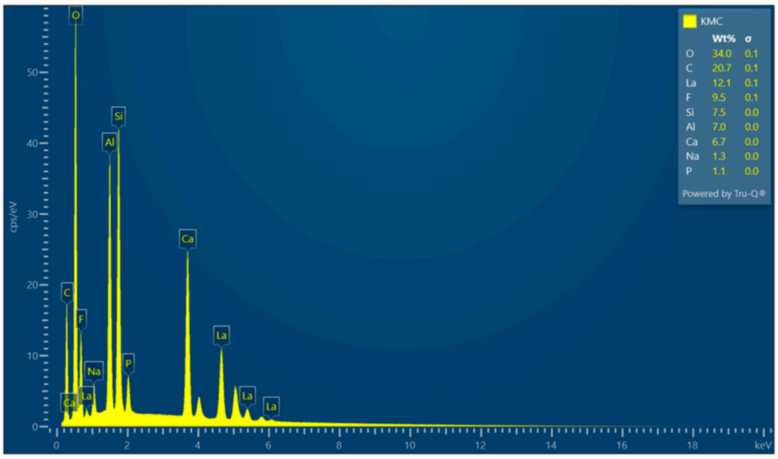

Figure S3. EDX spectrum of KM.

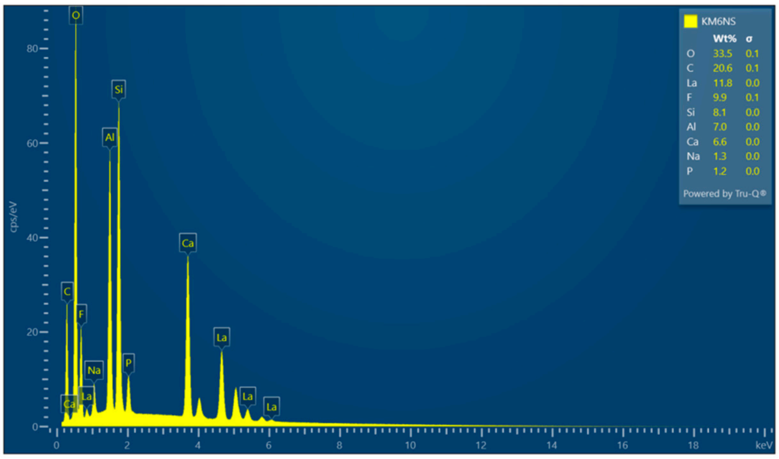

Figure S4. EDX spectrum of KM-6NS.

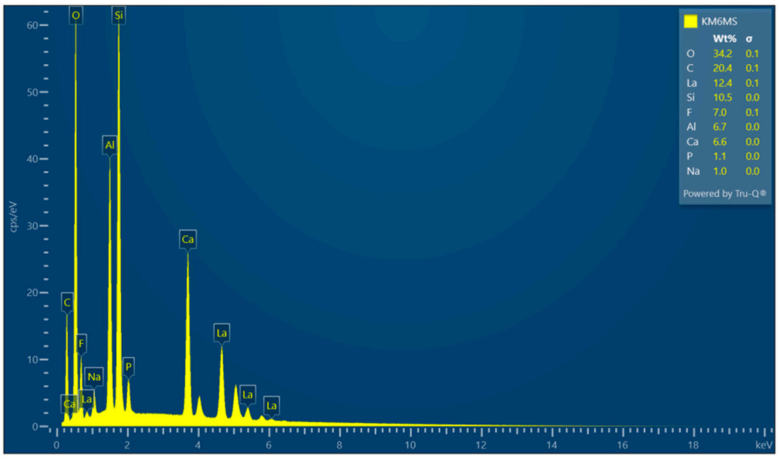

Figure S5. EDX spectrum of KM-6MS.

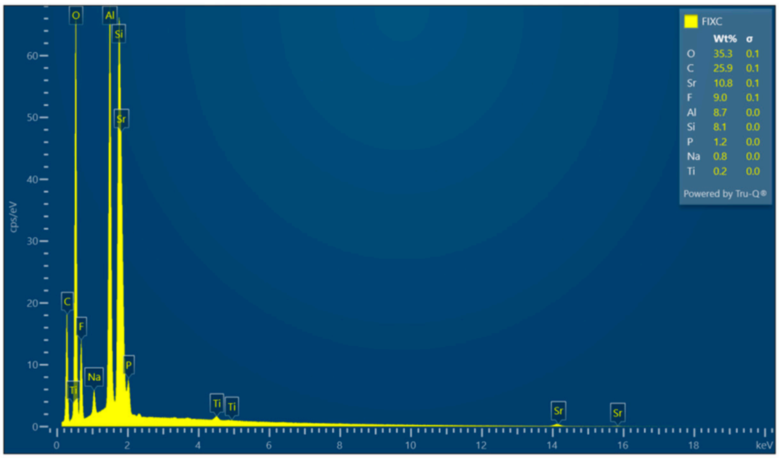

Figure S6. EDX spectrum of FIX.

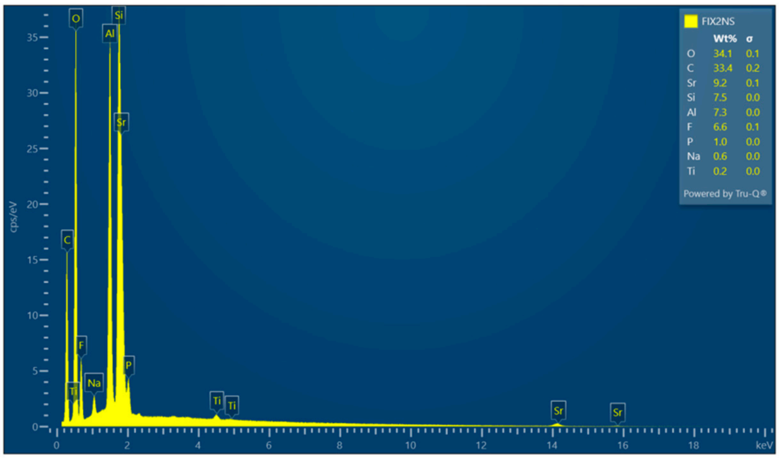

Figure S7. EDX spectrum of FIX-6NS.

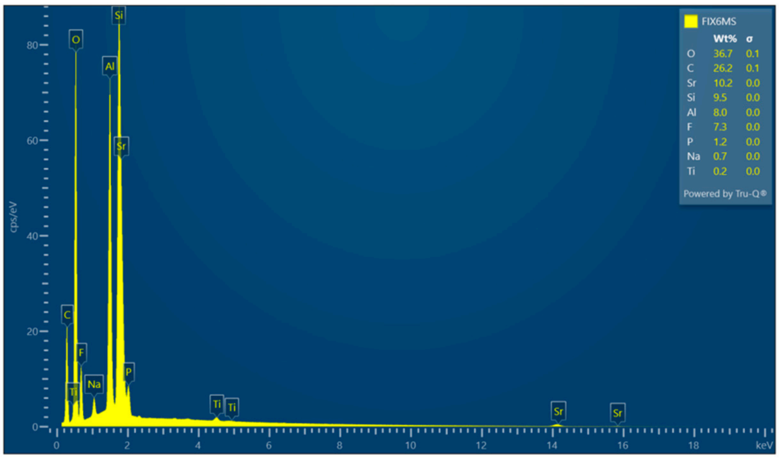

Figure S8. EDX spectrum of FIX-6MS.
